# Cuticular Antifungals in Spiders: Density- and Condition Dependence

**DOI:** 10.1371/journal.pone.0091785

**Published:** 2014-03-17

**Authors:** Daniel González-Tokman, Jasmin Ruch, Tamara Pulpitel, Fleur Ponton

**Affiliations:** 1 Departamento de Ecología Evolutiva, Instituto de Ecología, Universidad Nacional Autónoma de México, México D. F., México; 2 Biocenter Grindel & Zoological Museum, University of Hamburg, Hamburg, Germany; 3 Department of Biological Sciences, Macquarie University, Sydney, Australia; 4 School of Biological Sciences, The University of Sydney, Sydney, Australia; 5 Charles Perkins Centre, The University of Sydney, Sydney, Australia; USDA-Agricultural Research Service, United States of America

## Abstract

Animals living in groups face a high risk of disease contagion. In many arthropod species, cuticular antimicrobials constitute the first protective barrier that prevents infections. Here we report that group-living spiders produce cuticular chemicals which inhibit fungal growth. Given that cuticular antifungals may be costly to produce, we explored whether they can be modulated according to the risk of contagion (i.e. under high densities). For this purpose, we quantified cuticular antifungal activity in the subsocial crab spider *Diaea ergandros* in both natural nests and experimentally manipulated nests of varying density. We quantified the body-condition of spiders to test whether antifungal activity is condition dependent, as well as the effect of spider density on body-condition. We predicted cuticular antifungal activity to increase and body-condition to decrease with high spider densities, and that antifungal activity would be inversely related to body-condition. Contrary to our predictions, antifungal activity was neither density- nor condition-dependent. However, body-condition decreased with density in natural nests, but increased in experimental nests. We suggest that pathogen pressure is so important in nature that it maintains high levels of cuticular antifungal activity in spiders, impacting negatively on individual energetic condition. Future studies should identify the chemical structure of the isolated antifungal compounds in order to understand the physiological basis of a trade-off between disease prevention and energetic condition caused by group living, and its consequences in the evolution of sociality in spiders.

## Introduction

Living in groups is widespread and found in insects, spiders, birds and mammals, among other animals. Individuals that live in groups obtain benefits such as predator avoidance [1]–[4], foraging efficiency [5], [6], and enhanced reproductive success [5]. However, group living has associated costs: compared to solitary individuals or small groups, individuals in large groups can incur costs such as increased competition for resources [7]–[9]. Moreover, group-living animals are faced with the potential risk of accumulating pathogens that can spread more easily between group members [10], [11]. Therefore, group living can not only be costly in terms of competition between individuals but also in terms of pathogen defense and disease contagion [12]–[14].

One important cost derived from contagious diseases is the activation and use of immune responses. Immunity can be costly because of toxic byproducts of immune reactions or because it requires resources that are spent at the expense of other functions [15], [16]. To decrease these costs, some group-living animals modulate their investment in immune response according to the risk of infection [17]. Under crowded conditions (i. e., when contagion risk is high), some insects show more active immune system compared to organisms living in low densities [18], [19], this might allow them to be more resistant than individuals kept solitarily [19], [20]. Such density-dependent activation of immune responses can be interpreted as an adaptive strategy to decrease the costs associated with the maintenance and activation of immune defenses. A simpler strategy to deal with microorganisms is to avoid contagion, either via behavioural avoidance of infected individuals or places [21], [22], hygienic behaviour in the nest [23] or via chemical avoidance with antimicrobials on the skin or cuticle [24]–[26]. Despite incurring some cost, both behavioural and chemical protections can reduce the cost of activating the immune system once the pathogen has infected the host.

The subsocial crab spider *Diaea ergandros* lives in nests built from *Eucalyptus* leaves. Nests contain up to 70 spiderlings that are usually the offspring of a single female [27]. These nests persist several months and all spiders of the group communally enlarge the nest by attaching more leaves. The inside of these nests can be quite sealed, moldy and contain food debris [27], and therefore favors the development of pathogens with the risk of pathogenesis being elevated at increased conspecific density. Furthermore, infections can be particularly dangerous because group members are close relatives and there is thus low genetic variability that could result in more susceptible groups [14], [28]. Previous experimental research on *D. ergandros* shows that individuals in large groups build larger and more protective nests and survive better in the presence of a predator compared to small groups or singly kept spiders [29]. However, the influence of pathogen pressure on large spider groups might be higher but has not been explored yet.

The main aim of this study was to investigate whether *D. ergandros* spiders have developed density-dependent polyphenism pathogen defenses. Antifungal cuticular response in both natural nests and artificial nests of varying density was measured. Despite previous descriptions in different taxa, cuticular antifungal activity has not been described in spiders yet. Costs involved in the maintenance of cuticular antifungal activity were examined by measuring spiders' lipid body reserves. This study represents the first exploration of density dependence in preventive antifungal production within a spider species and evaluates its possible dependence on physiological condition (lipid reserves). We predict that antifungal protective activity will be a) present in crab spiders, b) a costly trait, dependent on physiological condition, and c) more intense with increasing nest density.

## Materials and Methods

### Ethics

No permits were required for the described study, which complied with all relevant regulations. The species used in these experiments (*Diaea ergandros*) is not an endangered or protected species under CITES regulations.

### Study species

The present study was carried out with the subsocial crab spider *Diaea ergandros* Evans, 1995 (Araneae: Thomisidae). Unlike other social and subsocial spiders, these spiders do not build webs and instead live in nests built from *Eucalyptus* leaves [30], [31]. Each nest consists mainly of a single mature female and her offspring, although migration between nests can occur [31], [32]. Even though spiders may migrate between nests, relatedness between nest mates is relatively high [31], [32]. Juveniles develop during 8–9 months after which they leave the nest [30]. For the present study we only used juvenile spiders of the instars 4 and 5 (see below). Spider nests were collected from *Eucalyptus* trees in June 2012 along the Lachlan Valley Way (34°47′5.02″S, 148°51′16.72″E; 34°32′37.32″S, 148°44′9.66″E) between Yass and Boorowa, NSW, Australia.

### Antifungal activity measurements

Antifungal activity was measured from the cuticle of spiders following a procedure modified from [24]. Since one spider did not provide enough sample of cuticular antifungal measurements (unpublished data), we used groups of five spiders for each sample. Spiders were anesthetized with carbon dioxide and washed with 2 mL of Ethanol 90% for five minutes to remove cuticular antifungals. Ethanol was evaporated from the sample with a rotary evaporator (25 mbar, 25°C). Under sterile conditions in a fume hood, each dry sample of spider extract was re-suspended in 125 μL of Luria Bertani (LB) broth and 100 μL of a culture of *Cordyceps bassiana* spores (2000–3000 spores/μL) in LB broth were added. Prior exploratory analysis varying spore concentration from 965–3580 spores/μL show no significant correlation with optical density after 24 h of fungal growth in LB broth (Pearson r = 0.16, P = 0.38, N = 15), showing that our assay is not sensitive to initial spore concentration. From each sample, 200 μL were placed in 96-well plates for measuring fungal growth as increments in optical density (OD) with time using a spectrophotometer (405 nm). Antifungal activity was measured as inhibition of spore germination after 24 hours in comparison with a positive control that consisted of a mixture of 100 μL of the *C. bassiana* culture and 100 μL of sterile LB broth. As a negative control (with no fungi, used to assure sterility during the assay), we used a mixture of 100 μL sample of spider extract in LB broth with 100 μL of sterile LB broth. At least two positive and one negative controls were used for each plate. The OD after 24 hours was considered the value of antifungal activity. Large OD values represent high fungi growth.

### Antifungal activity and energetic reserves under natural densities

Cuticular antifungal activity was measured in samples from 14 nests (LB broth with fungi and cuticular extracts; see below), 11 positive controls (LB broth with fungi) and 5 negative controls (LB broth without fungi). To examine the relationship between the level of antifungal activity in spider cuticles and the energetic body condition (lipid body reserves) under different densities, 20 nests that contained 12–61 spiders were used. Nest size was estimated by measuring length and width of each nest (±0.1 mm). Nest density was calculated as number of spiders/nest size. Due to contamination, antifungals could not be measured in 6 nests, and so sample size was reduced to 14 nests when antifungal activity was analyzed. For this part of the study, we only used juvenile spiders of 4^th^ and 5^th^ instars.

### Antifungal activity and energetic reserves under manipulated densities

In this experiment we tested if solitary spiders differed in their antifungal activity and body energetic reserves from their siblings kept in groups. For such purpose, individuals from selected nests were randomly allocated to one of two treatments: solitary and grouped spiders. Solitary spiders were kept individually and grouped spiders were kept in groups of 16 individuals in plastic transparent cups (100 mL) for 10 days under natural light and darkness regime. This controls for environmental and sanitary conditions that could be variable in natural nests that are probably exposed to different pathogens. Spiders were starved seven days before the experiment to get individuals with similar initial body condition at the start of the experiment. Once the experiment started, spiders were offered three meals consisting of one male and one female living *Drosophila melanogaster* per individual. After the 10 day period, five solitary or five grouped spiders from each nest were washed together in 90% Ethanol and antifungal activities were compared (see above). In this experiment we used a total of 10 nests of juveniles (ranging 27–85 spiders per nest) at the 4^th^ instar when antifungal activity and lipids were measured. Due to contamination, antifungals could not be measured in two of the 10 nests, leaving a total of eight nests for the analyses of antifungal activity.

As a measurement of individual body condition in both natural and artificial nests, we measured lipid body reserves [33]. Lipids were quantified as the difference in body dry weight before and after three 24 hour submersions in chloroform. We found that lipid reserves ranged from 0–6 mg in natural nests and 0–1.5 mg in artificial nests (see results). This can be explained by the age of the studied animals, considering that spiderlings grow with age: while natural nests comprised individuals of 4^th^ and 5^th^ instars, artificial nests included only individuals at the beginning of the 4^th^ instar.

### Statistical analyses

The relationship between nest density (number of spiders/cm^2^), antifungal activity and lipid reserves in nests taken directly from the field was examined using linear regressions. To test for the effect of density manipulation on antifungal activity and lipid contents, we used general linear mixed models with treatment (solitary or crowded) and original nest density (number of spiders) as explanatory variables as well as their interaction. The interaction between both covariates was tested but it was removed from the analysis for being non-significant (antifungals: P = 0.695, lipids: P = 0.634). Given that solitary and crowded treatments came from the same nest, nest ID was included as a random variable in the models. Relationships between antifungal activity levels and lipids were analyzed with Pearson correlations. The presence of outliers was examined with Cook's distances and variance homogeneity was tested with Fligner-Killeen tests [34]. All analyses were performed in R 2.10.0 [35].

## Results

**Figure 1:**
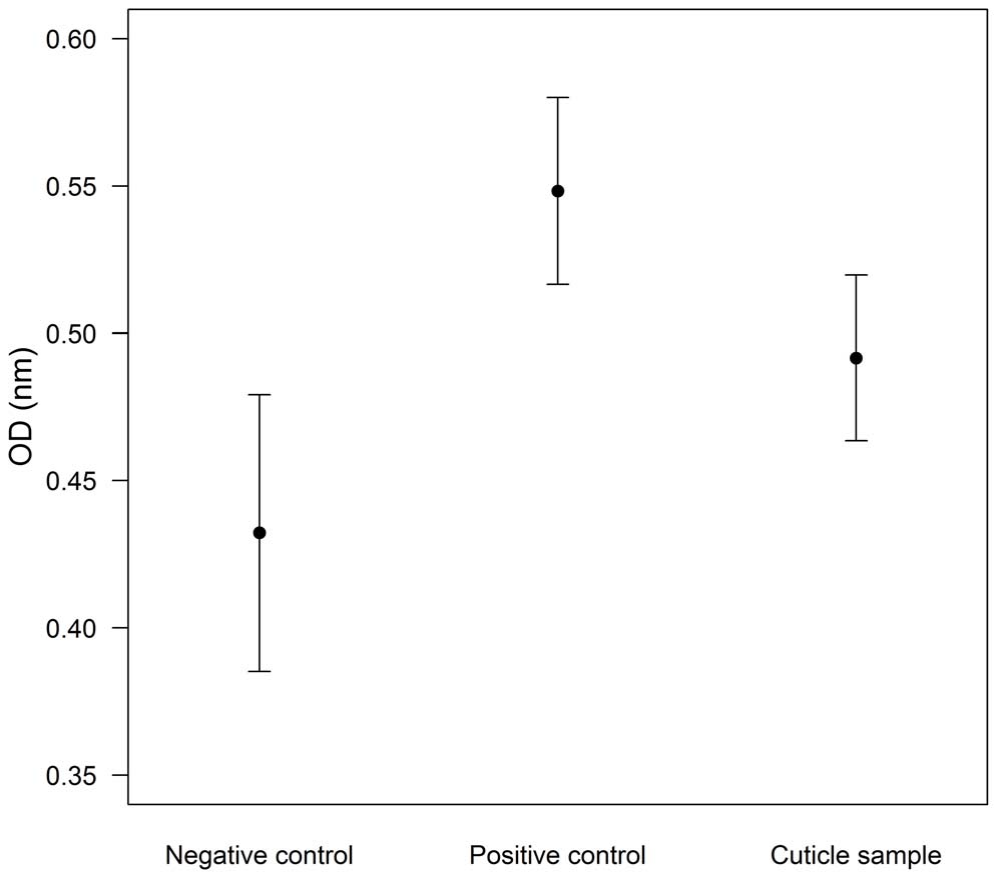
Effect of cuticular antifungals of *Diaea ergandros* spiders on fungal growth. Negative controls are cuticular samples without fungi, whereas positive controls are fungal cultures without cuticular samples. Bars represent means ±95% confidence intervals.

A washing of *D. ergandros* cuticles efficiently reduced fungal growth in a culture medium after 24 hours, showing that spiders possess antifungal activity in the cuticle that is effective against the fungal pathogen *Cordyceps bassiana* (ANOVA F_2,27_ = 9.416, P<0.001; Figure 1). A priori contrasts show that OD measurements of fungal growth in media with cuticle washings were significantly lower than OD of positive controls with *C. bassiana* (t = 2.744, P = 0.011) and significantly higher than negative controls (t = 2.228, P = 0.034); positive controls showed higher OD than negative controls (t = 4.202, P<0.001), confirming that there was no contamination in the culture medium.

**Figure 2:**
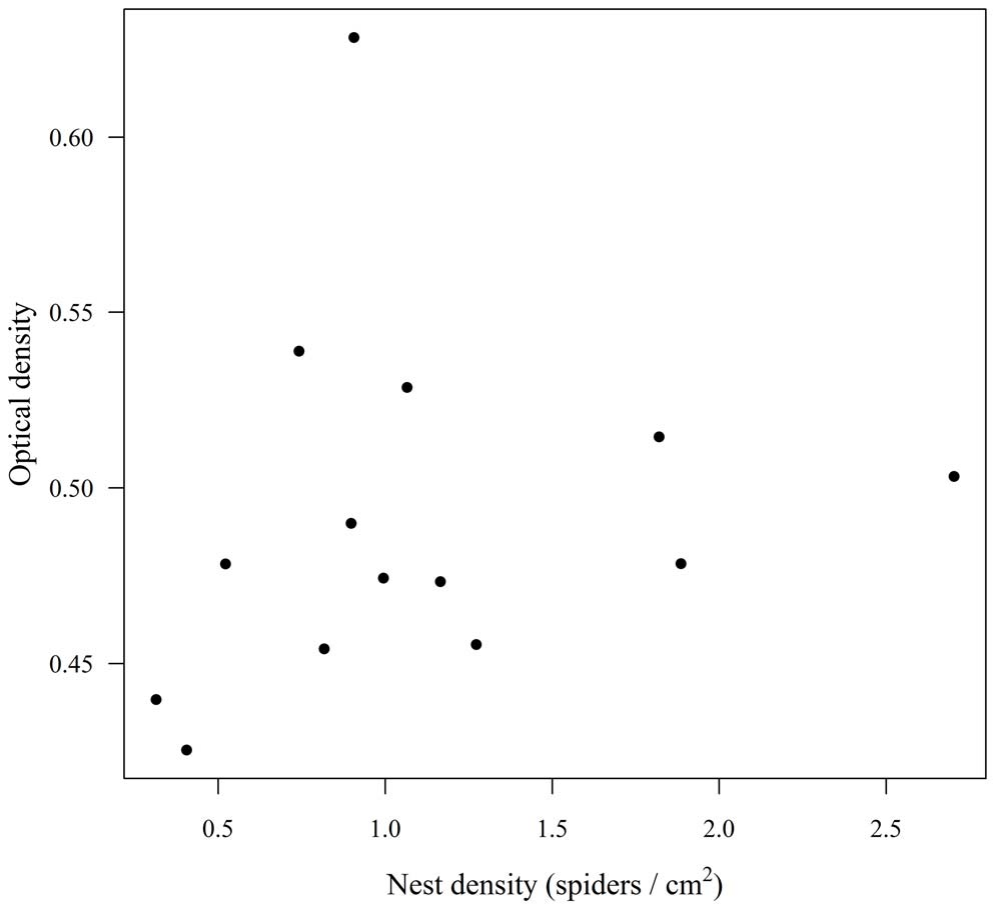
Relationship between nest density and antifungal activity in juvenile *Diaea ergandros* from natural nests (non-significant R^2^ = 0.035).

**Figure 3:**
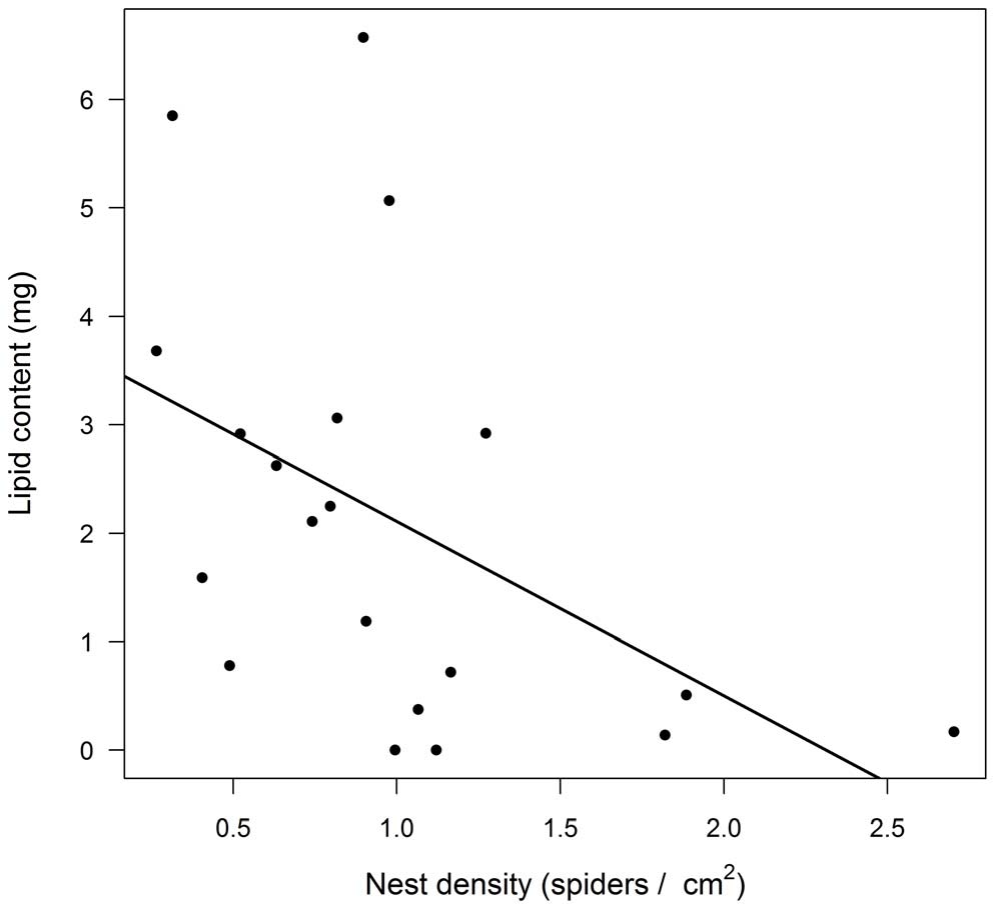
Relationship between nest density and lipid reserves in juvenile *Diaea ergandros* from natural nests (R^2^ = 0.229).

In nests taken from the field, there was no relationship between nest density and cuticular antifungal activity (R^2^ = 0.035, P = 0.524, N = 14; Figure 2). In addition, the intensity of cuticular antifungal activity was not correlated with the amount of lipid body reserves in individual spiders (Pearson R = −0.303, P = 0.293, N = 14). However, nest density and individual lipid content were negatively related (R^2^ = 0.229, P = 0.032, N = 20; Figure 3), meaning that spiders from high-density nests had a lower lipid content compared to spiders from low-density nests.

**Figure 4:**
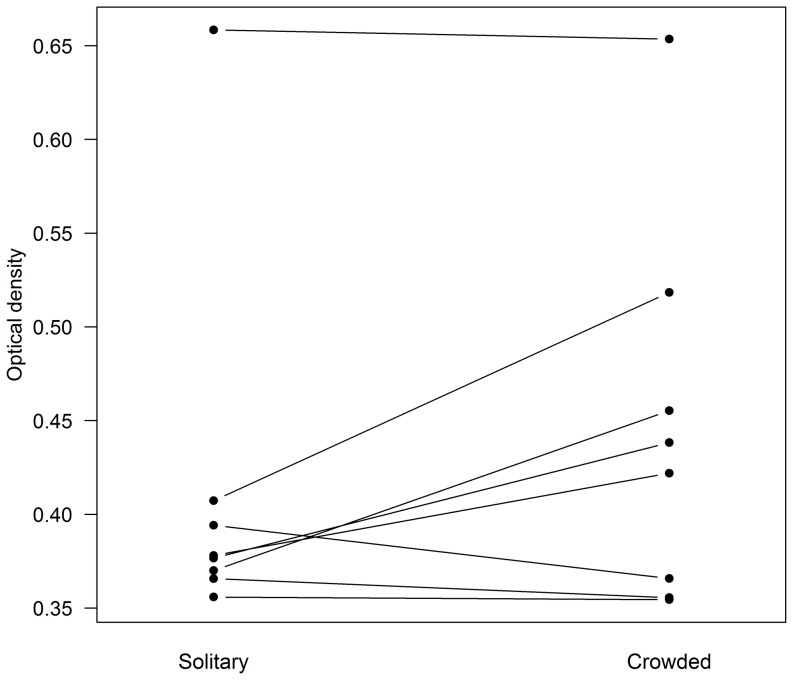
Antifungal activity in *Diaea ergandros* individuals that were experimentally kept crowded or solitary. Lines link individuals coming from the same nest.

**Figure 5:**
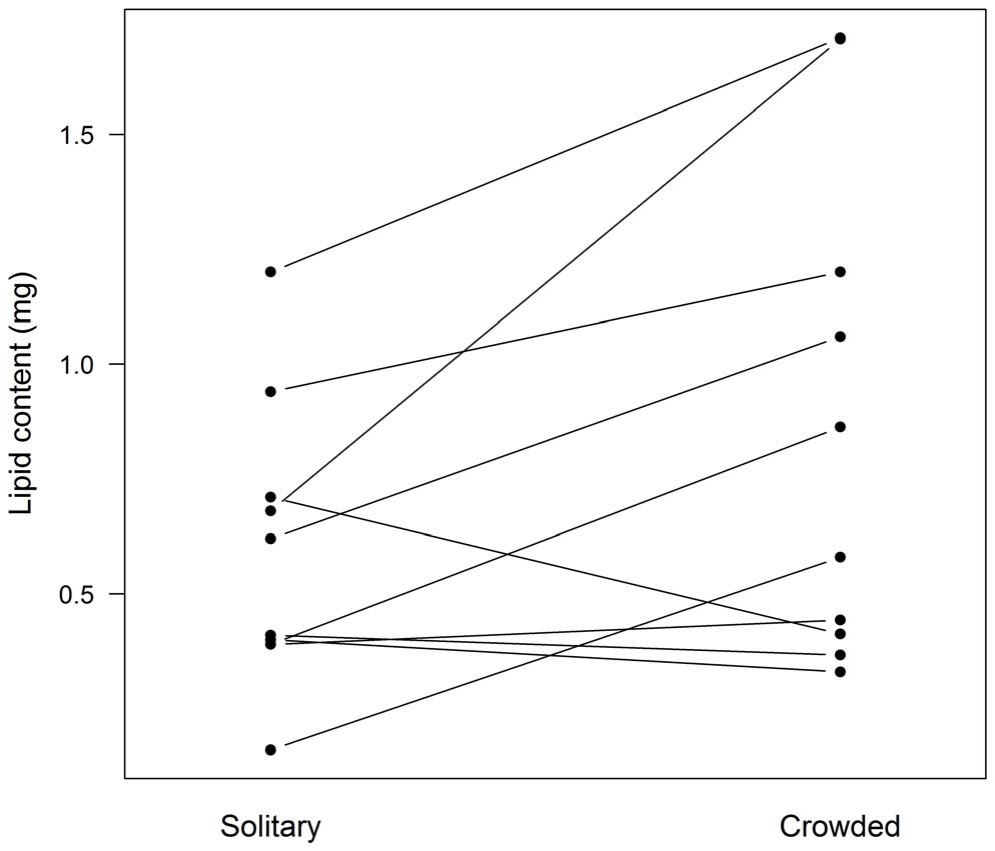
Lipid reserves in *Diaea ergandros* individuals that were experimentally kept crowded or solitary. Lines link individuals coming from the same nest.

In artificial nests, grouped spiders did not differ in antifungal activity from their solitary siblings (Mixed model F = 3.211, P = 0.116, N = 8; Figure 4). Initial spider density was controlled but was omitted from the final analysis for being non significant (F = 0.644, P = 0.453, N = 8). However, grouped spiders showed higher lipid contents than their solitary siblings (Mixed model F = 5.303, P = 0.047, N = 10; Figure 5). Initial spider density was marginally positively related to lipid reserves (F = 4.692, P = 0.062, N = 10). The intensity of cuticular antifungal activity was not correlated with the amount of lipid reserves in either solitary (Pearson R = −0.011, P = 0.980, N = 8) or grouped spiders (R = 0.196, P = 0.641, N = 8).

## Discussion

The present study represents the first evidence that spiders produce cuticular compounds that can reduce potential fungal infections. Cuticular antimicrobials have been described in insects such as ants, termites, wasps, bees, moths, thrips and whiteflies [24]–[26], [36], [37], and represent a first line defense against pathogens and parasites [38]. Our finding supports the idea that antimicrobial defense accompanies the evolution of sociality in animals living in large aggregations [13], [24].

A variety of antifungal compounds have been found in arthropod (particularly insect) cuticles, and they can be either secreted by the host itself or produced by symbiotic microorganisms [39]. These compounds can include free fatty acids, proteins (defensins), amides, aldehydes, terpenes, glucanase enzymes, chitinase and protease inhibitors, alkaloids and quinones [39], [40] which, together with cuticular melanization [41], [42], inhibit spore germination, hyphae growth or penetration into the body. In particular, melanization response [41], caprylic, valeric and nonanoic acid [26], monoterpenes [43] and salicylaldehyde [44] have shown to be effective against the same fungus used in the present study (*Cordyceps bassiana*) in lepidopterans and coleopterans. To our knowledge, there is no description of specific cuticular antifungals in spiders, but many of the compounds present in insects have ancient evolutionary origins and find homologies in other insects, nematodes and mammals [40], [45]–[47]; hence, similar compounds can also be present in spiders, contributing to the inhibition of fungal growth in our assays and probably against natural pathogens. Given that many of these compounds are soluble in ethanol, which was the solvent used for their extraction in the present study, our measure of antifungal activity probably includes the effect of several of these or related compounds. As a perspective of this work, investigating the chemical nature and the action spectrum of the cuticular compounds isolated in *D. ergandros*, as well as addressing to what extent they are synthesized by the host itself or by symbiotic microorganisms would give further insight into the study of antifungals in spiders.

The synthesis of the abovementioned cuticular antifungals [26], [36] certainly requires resources that are obtained from the host diet, such as amino-acids and fatty acids, and thus we predicted that only animals in good physiological condition would be able to produce effective antifungals. However, we found no relationship between energetic body-condition (lipid reserves) and intensity of antifungal activity. There are two possible explanations for this finding: (a) that these antifungals are not costly to produce and individuals in a range of conditions can maintain high levels, which seems unlikely given their chemical composition, or (b) that cuticular antifungals are so important in infection avoidance that individuals cannot allow to reduce their production. Given that living solitary can be costly in terms of foraging efficiency and predation risk [29], the latter interpretation can also explain why experimentally isolated individuals lost energetic condition compared to individuals kept in a group in our laboratory experiment.

Isolated individuals paid an energetic cost and not a cost in antifungal activity, probably because dietary restriction can be overcome [48] whereas loss of antifungals is too risky. If the same resources (e. g. amino-acids, fatty acids) are shared between cuticular antifungals and metabolic function, a trade-off between disease prevention and physiological condition may result [49]. As we ignore the plasticity of up- and down regulation of cuticular antifungals, we cannot discard the possibility that differences in antifungal activity could be detected in a long-term experiment.

We predicted that investment in antifungal protective activity would be higher in crowded nests, especially given the potentially higher risk of contagion that exists when there is high genetic relatedness among nest members [24], as in *D. ergandros*. However, we found no relationship between nest density and cuticular antifungal activity, neither in nature nor under laboratory conditions, suggesting that spiders constitutively express cuticular antifungal activity against the tested fungus. It is possible that cuticular antifungal activity, unlike immune response [17], [18], [50], is not a dynamic trait that can be regulated under varying risk of infection [21]. Other unmeasured components of spider defense, such as haemolymph immune response or melanization, might be adjusted under different densities, as occurs in other arthropods [17], [19], but this remains to be tested.

Despite the benefits of group living in terms of foraging, reproduction or predator avoidance [1], [4], [5], our results show that living at high densities is costly in terms of reducing energetic reserves. However, we only found this pattern under natural conditions, where food was not artificially supplemented and under natural predation risk. On the other hand, when density was manipulated in the laboratory, food was provided and predators were excluded, grouped spiders maintained higher lipid reserves than their solitary siblings. These contrasting results suggest that when animals are stressed (i. e. under natural conditions), living in large groups can be energetically disadvantageous, while when animals are not stressed (i. e. under laboratory conditions) living in groups is energetically beneficial. For example, nutrient availability under laboratory conditions could reduce cannibalism within nests affecting nest density [51], [52], but this idea remains to be tested.

In the present study we showed that living in large groups impacts on physiological condition of group members depending on the environmental conditions, presumably food availability. Despite energetic body condition being highly sensitive to group size, this was not the case for cuticular antifungal activity, which was not affected by nest density. The permanent pressure of pathogens on spider nests is likely to be responsible for the low plasticity of cuticular antifungal expression; if investment in pathogen protection needs to be constant, it can explain why energetic condition is compromised if resources are scarce. Future studies should formally evaluate the physiological basis of a potential trade off between lipid reserves and cuticular antifungals, and evaluate the importance of protective defense in the evolution of sociality.
